# Standardization of Dual-Energy CT Iodine Uptake of the Abdomen and Pelvis: Defining Reference Values in a Big Data Cohort

**DOI:** 10.3390/diagnostics14182051

**Published:** 2024-09-15

**Authors:** Ibrahim Yel, Christian Booz, Tommaso D’Angelo, Vitali Koch, Leon D. Gruenewald, Katrin Eichler, Aynur Gökduman, Davide Giardino, Michele Gaeta, Silvio Mazziotti, Eva Herrmann, Thomas J. Vogl, Scherwin Mahmoudi, Ludovica R. M. Lanzafame

**Affiliations:** 1 Division of Experimental Imaging, Department of Diagnostic and Interventional Radiology, University Hospital Frankfurt, 60596 Frankfurt, Germany; 2 Department of Diagnostic and Interventional Radiology, University Hospital Frankfurt, 60596 Frankfurt, Germany; 3 Diagnostic and Interventional Radiology Unit, BIOMORF Department, University of Messina, 98124 Messina, Italy; 4 Department of Radiology and Nuclear Medicine, Erasmus MC, 3015 CE Rotterdam, The Netherlands; 5 Institute of Biostatistics and Mathematical Modelling, Goethe University Frankfurt, 60596 Frankfurt, Germany

**Keywords:** contrast media, dual-energy computed tomography, iodine maps, abdominal imaging

## Abstract

**Background:** To establish dual-energy-derived iodine density reference values in abdominopelvic organs in a large cohort of healthy subjects. **Methods**: 597 patients who underwent portal venous phase dual-energy CT scans of the abdomen were retrospectively enrolled. Iodine distribution maps were reconstructed, and regions of interest measurements were placed in abdominal and pelvic structures to obtain absolute iodine values. Subsequently, normalization of the abdominal aorta was conducted to obtain normalized iodine ratios. The values obtained were subsequently analyzed and differences were investigated in subgroups defined by sex, age and BMI. **Results**: Overall mean iodine uptake values and normalized iodine ratios ranged between 0.31 and 6.08 mg/mL and 0.06 and 1.20, respectively. Women exhibited higher absolute iodine concentration across all organs. With increasing age, normalized iodine ratios mostly tend to decrease, being most significant in the uterus, prostate, and kidneys (*p* < 0.015). BMI was the parameter less responsible for variations in iodine concentrations; normal weighted patients demonstrated higher values of both absolute and normalized iodine. **Conclusions**: Iodine concentration values and normalized iodine ratios of abdominal and pelvic organs reveal significant gender-, age-, and BMI-related differences, underscoring the necessity to integrate these variables into clinical practice.

## 1. Introduction

Contrast-enhanced computed tomography (CT) is a fundamental tool in the evaluation of abdominal–pelvic pathologies. Contrast agent administration is often critical and increases diagnostic performance in the delineation and characterization of solid lesions, as well as in the assessment of disease extension [1]. The use of contrast media is essential to perform better tissue differentiation, determine its characteristics and evaluate its function [2]. The evaluation of tissue enhancement in conventional CT is based on the principle of X-ray attenuation, which is represented with a scale of CT numbers (Hounsfield Units) that are calibrated with reference to water, However, this method may lead to inaccuracies in the characterization of tissues due to the similar attenuation of different structures, which therefore does not allow a proper distinction [3].

The advent of dual-energy computed tomography (DECT), characterized by its utilization of more than one energy beam for image acquisition, facilitates the discrimination of tissue and material interactions across different energy levels, thereby enabling material decomposition and the extraction of augmented information from the acquired data sets [4]. The attenuation is mainly provided by two effects determined by the interaction of X-rays with matter: the photoelectric effect and the Compton effect. The photoelectric effect prevails at low energies, while the Compton effect is dominant at higher energy levels. The comparison of the attenuation differences of tissues at high and low energy levels therefore allows tissue characterization [5].

DECT offers a multitude of presentation options, such as virtual monoenergetic images (VMI), virtual non-contrast (VNC), and Z-effective reconstructions, which have demonstrated a profound impact on field imaging. Spectral datasets can also be used to generate iodine distribution maps that are rendered as quantitative, color-coded maps, which improve lesion conspicuity due to differences in iodine content between lesions and normal parenchyma. Iodine maps facilitate the detection and quantification of iodine within each image voxel, allowing for identification of subtle enhancements indicative of lesion presence [6,7,8,9]. The possibility of direct measurement of the contrast agent reduces the inter-individual variability for the evaluation of enhancement through a quantitative evaluation. Iodine maps can be used to differentiate between benign and malignant lesions and the possibility of direct iodine quantification suggested that iodine maps could be used as a surrogate for the assessment of tissue’s perfusion, as well as a potential predictive tool for evaluating the response to antiangiogenetic drugs in patients with highly vascular tumors [10,11,12]. So far, many studies have investigated the potential application of iodine maps in abdominopelvic imaging for the characterization of solid-organ lesions, inflammatory and traumatic diseases in the liver, pancreas, kidneys, adrenal glands, and bowel [13,14,15,16,17,18,19].

Despite the interest and application of DECT-derived iodine maps in clinical practice, the utilization of iodine density measurements for diagnostic purposes is predicated upon the establishment of normative reference values for physiological iodine distribution within the abdomen and pelvis to allow an adequate interpretation of the examinations. To date, the literature reveals a paucity of studies dedicated to determining these threshold values, highlighting a gap in standardization efforts. Consequently, the primary objective of this study is to investigate and establish DECT iodine uptake norms across a spectrum of healthy abdominal and pelvic organs, to facilitate a clearer differentiation between physiological and pathological concentrations of contrast medium for diagnostic purposes.

## 2. Materials and Methods

### 2.1. Ethical Considerations

This retrospective study strictly adhered to ethical guidelines and received approval from our institutional review board (Ref. Nr. 20-648). Informed consent requirements were waived due to the retrospective nature of the study.

### 2.2. Study Population

**Figure 1:**
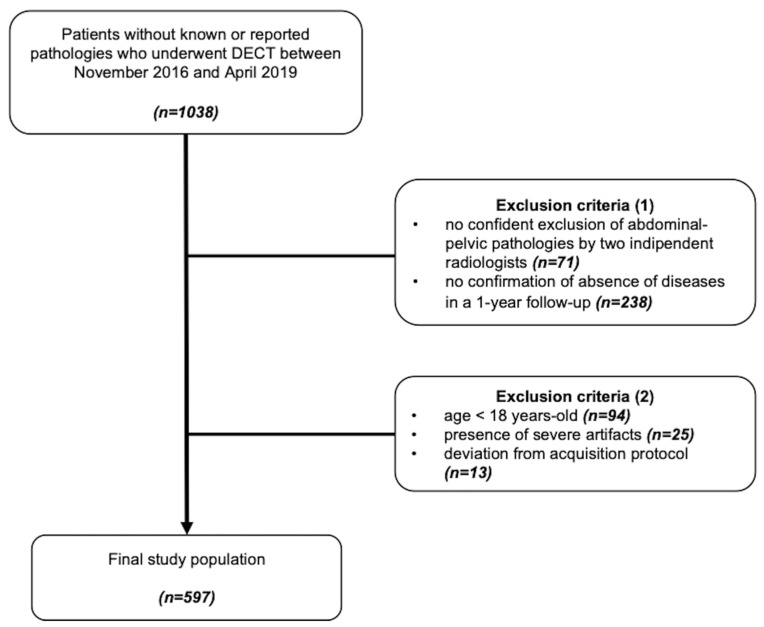
Study flowchart.

Consecutive patients, who underwent clinically indicated, contrast-enhanced DECT scans of the abdomen and pelvis for the exclusion of inflammatory disease, malignant lesions, traumas, or for the evaluation of abdominal pain between November 2016 and April 2019, were retrospectively enrolled in this study. The absence of abdominopelvic pathologies was carefully evaluated by two independent radiologists before inclusion, as well as the absence of symptoms or diseases in a 1-year follow-up (confirmed by anamnestic interviews, clinical examinations, ultrasound examinations, or cross-sectional imaging). Common laboratory values of all patients were also examined to ensure that they were in the normal range. Additional exclusion criteria were age < 18 years old, the presence of severe artifacts and/or deviation from the standardized acquisition protocol. Figure 1 shows the representation of the study population selection process.

### 2.3. DECT Imaging Technique

All examinations were performed on the same third-generation dual-source dual-energy CT scanner (SOMATOM Force, Siemens Healthcare, Forchheim, Germany) to ensure consistency across examinations. Patients were examined in supine position with intravenous administration of contrast agent after a delay of 80 s. Contrast media (Iomeron 350 mgI/mL, Bracco, Milan, Italy) was intravenously injected at a dose of 1.1 mL/kg and a flow rate of 3 mL/s through a superficial vein of the forearm. The CT examinations were all performed in craniocaudal scan direction using DE mode with different kilovoltage and tube current settings (90 kV and 190 mAs per rotation on tube A; Sn 150 kV with tin filter and 95 mAs per rotation on tube B). The rotation time was 0.5 s, collimation width 2 × 192 × 0.6 mm, and pitch 0.6.

### 2.4. Iodine Mapping and Uptake Measurements

Dual-energy analysis was performed by using the commercially available post-processing software Liver virtual non-contrast (Liver VNC) (version VB10B, Siemens Healthineers, Forchheim, Germany). Initially developed for iodine quantification in the liver, the Liver VNC software’s versatility has been demonstrated through its application in various anatomic regions [20]. Basis of its algorithm is a three-material-decomposition of iodine, fat, and tissue. The liver VNC application enables the visualization of iodine (contrast agent) concentrations by isolating the iodine content from the Hounsfield Unit value of any voxel and displaying of the pure iodine map as a colored overlay on the gray-scale image. The iodine slope was calculated automatically by the software at 90 keV and 150 keV, which refer to the X-ray energy levels used in the calculation. It is important to note that these values represent the effective energy levels for the dual-energy CT scans, not the X-ray tube voltages. The keV unit is commonly used to denote X-ray energy, crucial for calculating absorption in Hounsfield Units. While the X-ray source produces a continuous spectrum, the software selects these effective energy levels for analysis to optimize iodine quantification. Settings were left on default (Resolution: 2, Maximum [HU]: 3071, Iodine Ratio 3.46).

Circular dual-energy regions of interest (ROIs) were applied on axial iodine maps to assess iodine concentration (mg/mL) in liver, pancreas, spleen, adrenal glands, kidneys, lymph nodes, prostate, uterus and psoas muscles. The measurements were independently conducted by two board-certified radiologists to ensure accuracy and reproducibility. The size of the ROI measurements was adjusted based on the anatomical region being examined, ranging from 0.4 cm^2^ to 5.0 cm^2^, with careful placement to avoid the inclusion of surrounding tissue. In total, 32 ROIs per women and 34 ROIs per men were placed in each dataset to record absolute iodine concentration values.

In an effort to control for variations in patient-specific perfusion rates, and enhance the comparability of iodine concentrations, additional measurements were taken of the abdominal aorta’s iodine concentration. These measurements allowed for the normalization of data by calculating the iodine ratio:

$$Normalized\;iodine\;ratio=\frac{Absolute\;iodine\;value}{Aortic\;iodine\;value}$$

**Figure 2:**
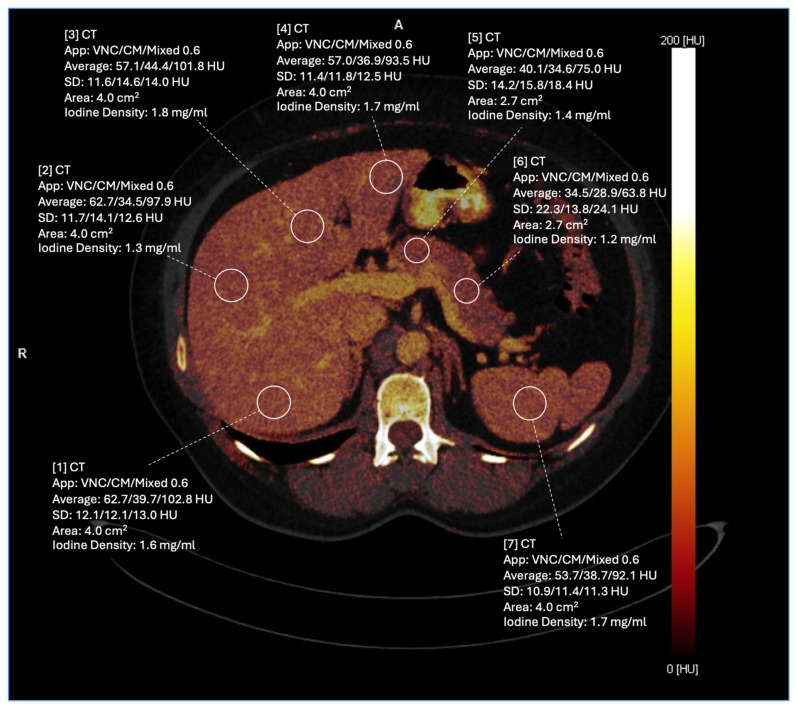
Exemplary demonstration of ROI placement on axial portal–venous phase iodine maps for liver, pancreas, and spleen.

The detailed list of the distribution of the iodine uptake measurements can be found in Table 1 and an example of ROI measurement placements within abdominal organs is shown in Figure 2.

### 2.5. Statistical Analysis

Statistical analysis was performed by using commercially available software (MedCalc Software Ltd., version 20, Ostend, Belgium). The Shapiro–Wilk test was employed to assess the distribution of data. Numeric values of continuous variables were given as means ± standard deviations (SD) for data following a normal distribution, or as medians and interquartile range (IQR) for non-normally distributed data. Categorical variables were expressed as counts or percentages. Unpaired *t*-test and analysis of variance (ANOVA) with Tukey multiple comparison post hoc tests were performed for normally distributed data. Mann–Whitney-U test and Kruskal–Wallis tests were applied in cases of non-normal distribution. A statistically significant difference was defined by a *p*-value < 0.05.

## 3. Results

### 3.1. Patient Population

The initial patient population included 1038 consecutive subjects of whom 441 were excluded according to the following criteria: no confident exclusion of abdominal–pelvic pathologies (*n* = 71), no confirmation of absence of diseases in a 1-year follow-up (*n* = 238), age < 18 years-old (*n* = 94), presence of severe artifacts (*n* = 25) and deviation from acquisition protocol (*n* = 13). The final study cohort consisted of 597 patients, predominantly Caucasian (*n* = 572), with a smaller representation from Asian (*n* = 19), Black (*n* = 9), and Hispanic (*n* = 7) backgrounds. The patient’s mean age was 63.3 ± 16.3 years. Subgroups were furtherly defined based on sex (335 men and 262 women), age (87 individuals aged 18–44 years, 212 aged 45–64 years, and 298 aged ≥ 65 years), and body mass index (BMI) (<25 [*n* = 251], 25–29.9 [*n* = 164] and ≥30 [*n* = 87] kg/m^2^). Patients’ demographic characteristics are specified in detail in Table 2. The mean CTDIvol and DLP were 8.3 ± 1.7 mGy and 496.5 ± 38.6 mGycm, respectively.

### 3.2. Overall Iodine Values

**Figure 3:**
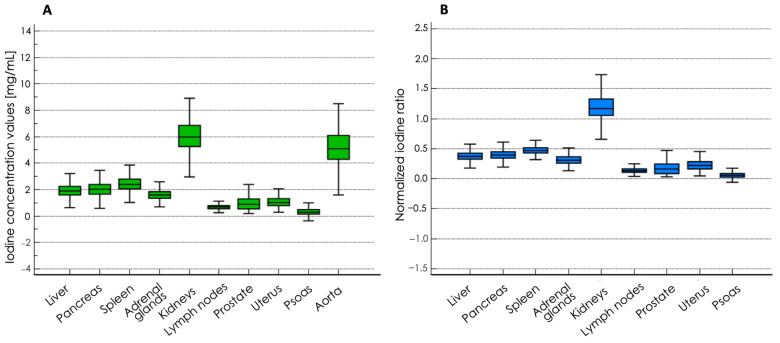
Box-and-whiskers plots showing iodine concentration values (**A**) and normalized iodine ratio (**B**) in abdominopelvic organs.

Among the abdominopelvic organs, the kidneys exhibited the highest absolute iodine concentration values (6.08 ± 1.32 mg/mL), followed by the spleen, which showed 2.46 ± 0.62 mg/mL of contrast agent uptake, whereas the psoas muscles registered the lowest (0.31 ± 0.27 mg/mL). After normalization with the iodine content values found in the descending aorta, the same trend persisted in the normalized iodine ratio, with 1.20 ± 0.24 mg/mL in the kidneys, 0.48 ± 0.09 mg/mL in the spleen and 0.06 ± 0.05 mg/mL in the psoas muscles. A comprehensive overview of the overall mean absolute iodine values and normalized iodine ratios is summarized in Table 3 and illustrated in Figure 3.

### 3.3. Impact of Sex

The comparison of iodine abdominal structures iodine uptake between sexes demonstrated higher absolute iodine concentrations in women across all abdominopelvic structures compared to men, with significant differences for all organs except lymph nodes (0.70 ± 0.18 vs. 0.72 ± 0.21 mg/mL—*p* = 0.58). On the other hand, men exhibited higher normalized iodine ratios, except in the psoas muscles (0.05 ± 0.05 vs. 0.07 ± 0.05 mg/mL—*p* < 0.0001). The pancreas (0.41 ± 0.09 vs. 0.40 ± 0.08 mg/mL—*p* = 0.31), and spleen (0.48 ± 0.09 vs. 0.48 ± 0.10 mg/L—*p* = 0.21) showed no significant sex-based differences in normalized iodine ratios. The complete list of absolute iodine uptake values and normalized iodine ratios for male and female is summarized in Table 4.

### 3.4. Impact of Age

**Figure 4:**
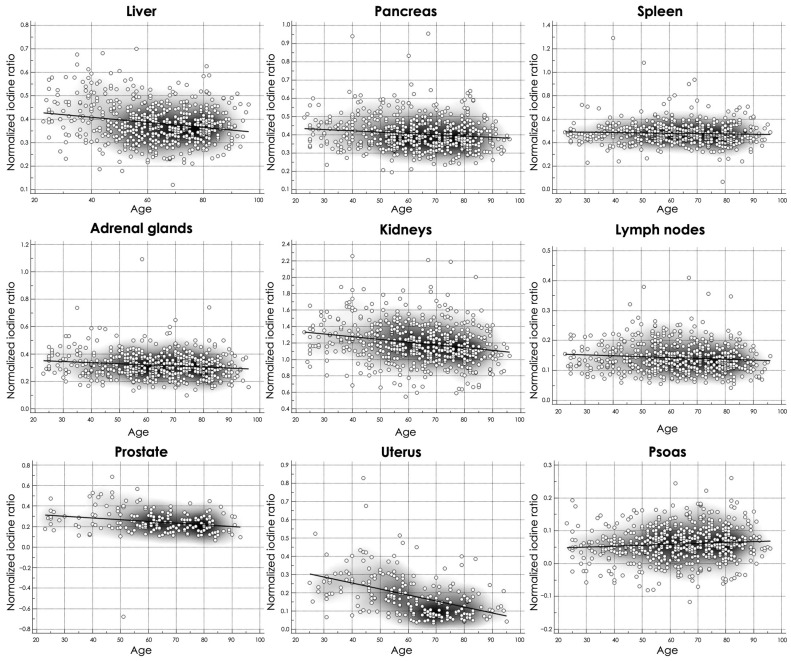
Scatter plots of normalized abdominopelvic iodine ratios values at different ages.

Comparing all three age groups, significant variances in absolute values were most pronounced between the youngest and oldest age groups (Table 5). Patients with age ≥ 65 years generally exhibited higher absolute values of uptake iodine for all organs than those aged 18–44 years, except the liver (1.98 ± 0.51 vs. 2.08 ± 0.51 mg/mL, *p* < 0.037), prostate (1.03 ± 0.37 vs. 1.18 ± 0.77 mg/mL, *p* = 0.0047) and uterus (0.72 ± 0.40 vs. 1.55 ± 0.99 mg/mL, *p* < 0.0001). Fewer significant differences have been reported between the old and middle-aged groups (liver, pancreas, spleen, uterus, and psoas—all *p* ≤ 0.033). The closest groups were the young and middle-aged groups, with only slight changes in iodine uptake for the liver (2.08 ± 0.51 vs. 1.88 ± 0.49 mg/mL—*p* = 0.0002), the lymph nodes (0.66 ± 0.17 vs. 0.73 ± 0.21 mg/mL—*p* = 0.0049), the prostate (1.18 ± 0.77 vs. 1.07 ± 0.33 mg/mL—*p* = 0.029) and the uterus (1.55 ± 0.99 vs. 2.13 ± 0.54 mg/mL—*p* = 0.019). However, when normalized iodine ratios were analyzed, the oldest group showed the most significant differences and lowest iodine ratios, except for the psoas muscles, compared to the two other age groups. Notably, both uterus and prostate iodine ratios showed a decline in their contrast medium enhancement with increasing age (Figure 4).

### 3.5. Impact of BMI

The BMI-based group analyses showed the least differences in both absolute and normalized iodine values (Table 6). Individuals with a normal BMI consistently showed higher absolute iodine concentrations compared to those categorized as overweight (25–29.9 kg/m^2^) or obese (≥30 kg/m^2^). Additionally, the obese group recorded lower iodine concentrations across all structures except for the uterus (1.03 ± 0.48 vs. 0.94 ± 0.62 mg/mL—*p* = 0.16) compared to overweight patients. No substantial differences were identified in normalized iodine ratios between overweight and obese patients, with the normal BMI group displaying higher normalized iodine uptake in the liver and pancreas, as summarized in Table 6.

## 4. Discussion

The aim of this study was to establish reference values for iodine concentration and perfusion ratios in abdominal–pelvic organs using contrast-enhanced DECT scans performed with dual-source technology in a large population of healthy patients.

DECT-derived iodine maps, with their capability for direct visualization and quantification of iodine distribution via color-coded overlays, offer a nuanced understanding of contrast medium concentration within tissues [21]. Although several parameters may affect the ability to obtain certain data concerning exact iodine uptake, such as the type of platform (e.g., dual-layer CT, dual-source CT, etc.) or patient characteristics, such as the overweight subjects for whom the quality of the images is lower due to increased noise from photon starvation, accuracy in iodine quantification has improved over time due to technological developments [9]. Third-generation dual-source CT systems proved to accurately quantify iodine concentration in phantom studies with median measurement errors of −4.0% (IQR −6.0, −2.8%) at 150 Sn/90 kVp, as reported by Pelgrim et al. [6]. While previous research has explored the diagnostic utility of iodine concentration maps for various abdominal pathologies, focusing on individual organs, in particular liver, pancreas, kidneys, and adrenal glands, comprehensive standardization of normal iodine uptake values remains essential to distinguish specifically in each organ the presence of normal or pathological concentrations of contrast agent within several structures [20,22,23,24,25,26,27,28]. In addition, such standardization must account for inter-individual variability, including differences attributable to sex, age, and BMI.

Our findings contribute to the body of knowledge by delineating iodine concentration reference values across a broad cohort of healthy individuals. The included individuals had received clinically indicated CT examinations for the exclusion of inflammatory changes, exclusion of tumorous pathologies, after trauma, or for the evaluation of abdominal pain, which in each case were not confirmed by CT-imaging and laboratory results and showed no recurrence after a 1-year interval.

On an absolute basis, the kidneys and spleen exhibited the highest iodine concentrations and normalized perfusion ratios, whereas the psoas muscles demonstrated the lowest values. In contrast to literature reports suggesting higher iodine values in healthy lymph nodes, our study presents more modest values, underscoring potential variations in iodine uptake across different anatomical regions and methodologies. For instance, Sauter et al. analyzed iodine content in lymph nodes in 99 patients and found values of 2.09 mg/mL for neck, 1.24 mg/mL for axilla, and 1.11 mg/mL for groin, showing significantly higher iodine concentrations in lymph nodes compared to our findings [29]. In another study from Martin et al., in which the aim was to quantify iodine and fat to discriminate malignant abdominal lymphoma from lymph node metastasis, the authors reported higher absolute iodine density values in healthy lymph nodes (2.4 ± 0.8 mg/mL) compared to our study [30].

On the other hand, two studies by Martin et al. reported iodine values that align with our findings. The first study aimed to evaluate differences in iodine concentration among healthy adrenal glands, adrenal adenomas, and adrenal metastases, revealing healthy adrenal gland values of 1.7 ± 0.6 mg/mL, comparable to the 1.61 ± 0.43 mg/mL observed in our research, which significantly differed from adrenal adenomas and adrenal metastases (1.3 ± 0.4 mg/mL and 3.2 ± 1.4 mg/ml, respectively; all *p* ≤ 0.003) [15]. In the second paper, the authors determined absolute iodine values of 1.8 ± 0.3 mg/mL for patients with acute pancreatitis and 2.7 ± 0.7 mg/mL for normal pancreatic parenchyma, which are in line with our findings (2.07 ± 0.53 mg/mL) [20], further supporting the consistency of our results with those of the established literature.

However, none of these studies performed the calculation of iodine perfusion ratio to normalize iodine uptake values according to the aortic iodine concentration, nor were the inter-individual differences analyzed. Normalizing iodine uptake values may be crucial to minimizing variations in tissue contrast enhancement caused by inter-individual differences and the effects of different contrast agents and injection protocol. Our analysis extends beyond the mere establishment of reference values by examining the impact of sex, age, and BMI on iodine distribution. Significantly, we observed marked sex-related differences in iodine concentrations across all organs with higher absolute iodine concentrations in women and higher iodine perfusion ratio in men. Furthermore, we detected a general trend of decreasing iodine density in abdominopelvic organs with advancing age, particularly notable in the uterus. This aligns with the recent stud of Zopfs et al., which tries to identify reference values of iodine density and perfusion ratio using a dual-layer spectral CT scanner. The authors observed a similar trend in the perfusion ratio of the uterus. For the prostate, they noted that the values exhibited a decreasing trend until middle age, after which they remained stable [31]. Beck et al. investigated iodine concentration range for healthy liver parenchyma in the portal–venous phase with a dual-layer CT platform and determined the impact of gender and age in contrast medium uptake. Their results showed a noteworthy difference in the absolute iodine concentration of the liver parenchyma between men and women. Women’s absolute iodine concentration was 2.37 ± 0.48 mg/mL, whereas for men it was 1.91 ± 0.47 mg/mL; these values are similar to our findings of 2.11 ± 0.56 mg/mL and 1.84 ± 0.43 for female and male, respectively. This feedback further supports the need to create gender-specific ranges for the proper quantitative evaluation of dual-energy iodine maps. Age did not influence the absolute iodine concentration of the liver parenchyma, but on the other hand a correlation was found after normalization with the portal vein and the mixed blood (portal vein and aorta) normalized iodine concentration [32]. BMI, on the other side, was not seen to be a determining factor for contrast agent concentration in various tissues, with some differences found mainly between patients with normal weight and the obese.

Our study acknowledges several limitations beyond its retrospective design. Firstly, it is constrained by the specific acquisition and contrast medium administration protocol employed, as well as using a vendor-specific CT system, potentially limiting applicability across different DECT technologies. Despite this, research indicates a generally high level of accuracy in iodine quantification across various DECT scanners, even in the presence of measurement variations [33]. Secondly, our analysis was confined to portal–venous phase scans; hence, employing different scan phases might yield divergent outcomes and variations in iodine concentration values. Thirdly, the precision of ROI measurements in smaller anatomical areas could have introduced some inaccuracies. Specifically, iodine measurements for lymph nodes were conducted only in instances where they were sufficiently large to accommodate a ROI of at least a 4 mm^2^ size. Furthermore, our study did not incorporate specialized laboratory tests, which limits our ability to identify potential deviations in more specific laboratory parameters. Lastly, while our study cohort was sizeable, it predominantly comprised Caucasian patients, underscoring the need for future research to include a more ethnically diverse participant base to ensure the generalizability of the findings. In addition, the sub-groups of patients analyzed were not perfectly homogeneous for the prevalence of patients over 65 years in age categorization and with BMI below 25 kg/m^2^.

## 5. Conclusions

In conclusion, this study establishes foundational reference values for iodine concentration and perfusion ratios in healthy abdominal–pelvic structures, emphasizing the necessity of considering sex, age, and BMI variations in clinical assessments. Future investigations should aim to overcome the limitations noted, particularly through the inclusion of diverse patient populations and exploration of additional DECT technologies and scanning phases.

## Figures and Tables

**Table 1:** Region of interest localization per organ.

Structure	Number of ROI	Localization
Liver	9	Liver segments (I–VIII)
Pancreas	3	Head, body and tail
Spleen	3	Medial, central, and lateral part
Adrenal glands	2	Left and right adrenal gland
Kidneys	6	Each side: upper pole, center, lower pole
Lymph nodes	3	Mesenteric, retroperitoneal and inguinal
Prostate	4	Peripheral zone (2), transition zone and central zone
Uterus	2	Anterior and posterior wall
Psoas muscles	2	Each side at the height of the third lumbar vertebrae
Aorta	1	Abdominal aorta

ROI: region of interest.

**Table 2:** Demographic characteristics of study population.

	Total	Men	Women
Number	597	335	262
Age (years)	63.3 ± 16.3	63.3 ± 16.4	63.4 ± 16.3
18–44 years (*n*)	87	49	38
45–64 years (*n*)	212	120	92
≥65 years (*n*)	298	166	132
BMI (kg/m^2^)	25.8 ± 5.3	25.9 ± 4.6	25.7 ± 6.1
<25 kg/m^2^ (*n*)	251	134	117
25–29.9 kg/m^2^ (*n*)	164	110	54
≥30 kg/m^2^ (*n*)	87	42	45

**Table 3:** Absolute iodine concentration and normalized iodine ratio values for the different structures of abdomen and pelvis.

Structure	Absolute Iodine Concentration (mg/mL)	Normalized Iodine Ratio
Liver	1.96 ± 0.51	0.38 ± 0.08
Pancreas	2.07 ± 0.53	0.41 ± 0.09
Spleen	2.46 ± 0.62	0.48 ± 0.09
Adrenal glands	1.61 ± 0.43	0.32 ± 0.09
Kidneys	6.08 ± 1.32	1.20 ± 0.24
Lymph nodes	0.71 ± 0.20	0.14 ± 0.05
Prostate	1.07 ± 0.44	0.23 ± 0.12
Uterus	1.01 ± 0.66	0.18 ± 0.12
Psoas	0.31 ± 0.27	0.06 ± 0.05
Aorta	5.20 ± 1.22	1

**Table 4:** Absolute iodine concentration and normalized iodine ratio values for men and women (* asterisk indicates statistical significance).

Absolute Iodine Concentration (mg/mL)			
Structure	Sex		*p*-Value
	Men	Women	
Liver	1.84 ± 0.43	2.11 ± 0.56	<0.0001 *
Pancreas	1.91 ± 0.46	2.28 ± 0.54	<0.0001 *
Spleen	2.26 ± 0.50	2.72 ± 0.65	<0.0001 *
Adrenal glands	1.52 ± 0.39	1.72 ± 0.47	<0.0001 *
Kidneys	5.67 ± 1.17	6.61 ± 1.33	<0.0001 *
Lymph nodes	0.70 ± 0.18	0.72 ± 0.21	0.5809
Prostate	1.06 ± 0.49	-	n/a
Uterus	-	1.01 ± 0.66	n/a
Psoas	0.23 ± 0.24	0.42 ± 0.28	<0.0001 *
Aorta	4.74 ± 1.05	5.77 ± 1.19	<0.0001 *
**Normalized Iodine Ratio**			
**Structure**	**Sex**		***p*-Value**
	**Men**	**Women**	
Liver	0.39 ± 0.08	0.37 ± 0.08	<0.0002 *
Pancreas	0.41 ± 0.09	0.40 ± 0.08	0.3083
Spleen	0.48 ± 0.09	0.48 ± 0.10	0.2196
Adrenal glands	0.33 ± 0.09	0.30 ± 0.09	<0.0001 *
Kidneys	1.23 ± 0.25	1.17 ± 0.25	<0.0079 *
Lymph nodes	0.15 ± 0.05	0.13 ± 0.04	<0.0001 *
Prostate	0.24 ± 0.14	-	n/a
Uterus	-	0.18 ± 0.12	n/a
Psoas	0.05 ± 0.05	0.07 ± 0.05	<0.0001 *

n/a: not available

**Table 5:** Absolute iodine concentration and normalized iodine ratio for men and women groups (* asterisk indicates statistical significance).

Absolute Iodine Concentration (mg/mL)						
Structure	Age			*p*-Values		
	18–44	45–64	≥65	18–44 vs. 45–64	45–64 vs. ≥65	18–44 vs. ≥65
Liver	2.08 ± 0.51	1.88 ± 0.49	1.98 ± 0.51	0.0002 *	0.0139 *	0.0365 *
Pancreas	2.08 ± 0.62	2.02 ± 0.53	2.10 ± 0.49	0.7327	0.0320 *	0.1880
Spleen	2.33 ± 0.66	2.42 ± 0.58	2.53 ± 0.62	0.1316	0.0330 *	0.0023 *
Adrenal glands	1.60 ± 0.47	1.57 ± 0.42	1.64 ± 0.43	0.6340	0.0525	0.4461
Kidneys	6.21 ± 1.23	5.95 ± 1.19	6.14 ± 1.43	0.1015	0.1369	0.5559
Lymph nodes	0.66 ± 0.17	0.73 ± 0.21	0.71 ± 0.19	0.0049 *	0.3069	0.0273 *
Prostate	1.18 ± 0.77	1.07 ± 0.33	1.03 ± 0.37	0.0290 *	0.2443	0.0047 *
Uterus	1.55 ± 0.99	2.13 ± 0.54	0.72 ± 0.40	0.0192 *	<0.0001 *	<0.0001 *
Psoas	0.25 ± 0.22	0.29 ± 0.27	0.35 ± 0.28	0.2839	0.0136 *	0.0029 *
Aorta	4.92 ± 1.28	5.04 ± 1.20	5.39 ± 1.19	0.4118	0.0002 *	0.0010 *
**Normalized Iodine Ratio**						
**Structure**	**Age**			***p*-Values**		
	**18–44**	**45–64**	**≥65**	**18–44 vs. 45–64**	**45–64 vs. ≥65**	**<45 vs. ≥65**
Liver	0.44 ± 0.10	0.38 ± 0.08	0.37 ± 0.07	<0.0001 *	0.3645	<0.0001 *
Pancreas	0.43 ± 0.10	0.41 ± 0.09	0.40 ± 0.08	0.0296 *	0.2872	0.0030 *
Spleen	0.49 ± 0.12	0.48 ± 0.08	0.48 ± 0.09	0.5801	0.1908	0.6861
Adrenal glands	0.34 ± 0.10	0.32 ± 0.09	0.31 ± 0.09	0.2766	0.1756	0.0412 *
Kidneys	1.31 ± 0.26	1.21 ± 0.22	1.17 ± 0.29	0.0025 *	0.0152 *	<0.0001 *
Lymph nodes	0.14 ± 0.04	0.15 ± 0.05	0.14 ± 0.05	0.4252	0.0054 *	0.1975
Prostate	0.27 ± 0.23	0.24 ± 0.09	0.21 ± 0.08	0.0112 *	0.0025 *	<0.0001 *
Uterus	0.27 ± 0.24	0.21 ± 0.12	0.13 ± 0.07	0.0074 *	<0.0001 *	<0.0001 *
Psoas	0.05 ± 0.05	0.06 ± 0.05	0.06 ± 0.05	0.2721	0.0986	0.0201 *

**Table 6:** Absolute iodine concentration and normalized iodine ratio values for age groups (* asterisk indicates statistical significance).

Absolute Iodine Concentration (mg/mL)						
Structure	BMI			*p*-Values		
	<25	25–29.9	≥30	<25 vs. 25–29.9	25–29.9 vs. ≥30	<25 vs. ≥30
Liver	2.11 ± 0.53	1.85 ± 0.47	1.78 ± 0.42	<0.0001 *	0.4983	<0.0001 *
Pancreas	2.20 ± 0.57	1.96 ± 0.52	1.94 ± 0.42	<0.0001 *	0.9103	0.0002 *
Spleen	2.56 ± 0.66	2.35 ± 0.60	2.29 ± 0.47	0.0005 *	0.7232	0.0004 *
Adrenal glands	1.65 ± 0.44	1.59 ± 0.43	1.53 ± 0.48	0.0571	0.3631	0.0078 *
Kidneys	6.21 ± 1.44	6.03 ± 1.28	5.77 ± 1.09	0.2820	0.0947	0.0103 *
Lymph nodes	0.72 ± 0.21	0.72 ± 0.19	0.66 ± 0.17	0.8681	0.0230 *	0.5198
Prostate	1.10 ± 0.41	1.07 ± 0.35	0.91 ± 0.74	0.8567	0.1339	0.1212
Uterus	1.08 ± 0.75	0.94 ± 0.62	1.03 ± 0.48	0.1998	0.1566	0.8304
Psoas	0.34 ± 0.29	0.28 ± 0.26	0.27 ± 0.27	0.0627	0.4150	0.0177 *
Aorta	5.38 ± 1.23	4.97 ± 1.22	4.89 ± 1.14	0.0006 *	0.5828	0.0012 *
**Normalized Iodine Ratio**						
**Structure**	**BMI**			***p*-Values**		
	**<25**	**25–29.9**	**≥30**	**<25 vs. 25–29.9**	**25–29.9 vs. ≥30**	**<25 vs. ≥30**
Liver	0.40 ± 0.08	0.38 ± 0.08	0.37 ± 0.09	0.0112 *	0.4514	0.0064 *
Pancreas	0.42 ± 0.09	0.40 ± 0.08	0.41 ± 0.10	0.0685	0.9420	0.1070
Spleen	0.48 ± 0.10	0.48 ± 0.08	0.48 ± 0.09	0.4825	0.6617	0.3090
Adrenal glands	0.31 ± 0.08	0.33 ± 0.9	0.33 ± 0.12	0.1373	0.2548	0.9023
Kidneys	1.18 ± 0.24	1.24 ± 0.21	1.22 ± 0.29	0.0010 *	0.1668	0.4080
Lymph nodes	0.14 ± 0.04	0.15 ± 0.06	0.14 ± 0.04	0.0531	0.3803	0.5198
Prostate	0.23 ± 0.09	0.25 ± 0.09	0.21 ± 0.24	<0.0001 *	0.3357	0.8826
Uterus	0.19 ± 0.12	0.17 ± 0.13	0.21 ± 0.10	0.0906	0.0968	0.6298
Psoas	0.06 ± 0.05	0.06 ± 0.05	0.05 ± 0.06	0.7878	0.1167	0.1490

## Data Availability

The data presented in this study are available on request from the corresponding author.
